# Assessing the effectiveness of a sexual and reproductive health and rights training programme in changing healthcare practitioners’ attitudes and practices in low-income countries

**DOI:** 10.1080/16549716.2023.2230814

**Published:** 2023-07-17

**Authors:** Gilbert Tumwine, Per-Olof Östergren, Christina Gummesson, Anette Agardh

**Affiliations:** a Department of Clinical Sciences, Social Medicine and Global Health, Lund University, Malmö, Sweden; b Obstetrics and Gynecology Department, St. Francis Hospital Nsambya, Kampala, Uganda; c Faculty of Medicine, Lund University, Lund, Sweden

**Keywords:** Healthcare practitioners, SRHR attitudes, SRHR behaviours, SRHR knowledge seeking behaviour, SRHR knowledge

## Abstract

**Introduction:**

In low-income countries the utilisation of sexual and reproductive health and rights (SRHR) services is influenced by healthcare practitioners’ knowledge, attitudes and practices. Despite awareness of the potential problems due to ingrained biases and prejudices, few approaches have been effective in changing practitioners’ knowledge, attitudes and practices concerning SRHR in low-income countries.

**Objectives:**

1) To assess whether participating in an SRHR international training programme (ITP) changed healthcare practitioners’ SRHR knowledge, SRHR attitudes and SRHR practices and 2) examine associations between trainees’ characteristics, their SRHR work environment and *transfer of training*.

**Methods:**

A pre- and post-intervention study, involving 107 trainees from ten low-income countries, was conducted between 2017 and 2018. Paired samples t-test and independent samples t-test were used to assess differences between trainees’ pre- and post-training scores in self-rated SRHR knowledge, attitudes, knowledge seeking behaviour and practices. Linear regression models were used to examine association between trainees’ baseline characteristics and post-training attitudes and practices.

**Results:**

Trainees’ self-rated scores for SRHR knowledge, attitudes and practices showed statistically significant improvement. Baseline high SRHR knowledge was positively associated with improvements in attitudes but not practices. High increases in scores on knowledge seeking behaviour were associated with higher practice scores. No statistically significant associations were found between scores that measured changes in SRHR knowledge, attitudes and practices.

**Conclusion:**

The findings indicate that the ITP was effective in improving trainees’ self-rated scores for SRHR knowledge, attitudes and behaviours (practices). The strongest association was found between improvement in SRHR knowledge seeking behaviour and the improvement in SRHR practices. This suggests that behaviour intention may have a central role in promoting fair open-minded SRHR practices among healthcare practitioners in low-income countries.

## Introduction

The 2030 Agenda for Sustainable Development and the initiative for Universal Health Coverage highlight the importance of sexual and reproductive health (SRH) for human development. In many low- and middle-income countries (LMICs) access to SRH is often inadequate and of poor quality [1]. The quality of health services impacts on utilisation and health outcomes. It is estimated that almost six out of ten deaths in LMICs may be related to poor quality health services. People living in disadvantaged conditions often receive poorer quality services than those who are relatively more advantaged [2]. High-quality healthcare is described as non-discriminatory, equitable and responsive to user needs and experiences. The Lancet Global Health Commission on High-Quality Health Systems in the Sustainable Development Goals [2,3] suggests that in order to improve and maintain health consistently, health systems should be reliable, dependable and mindful of provider-client interactions and client experiences.

Healthcare practitioners’ attitudes and behaviours influence client experiences and contribute to the perception of quality of care. Negative attitudes and practices by healthcare providers alienate service users and impact on their utilisation. This is particularly true when providers must deal with sensitive issues such as sexual and reproductive health and rights (SRHR) [1,4–6]. The World Health Organization (WHO) designates the ‘health workforce’ as one of the six essential building blocks for strengthening healthcare systems and highlights the significance of a workforce with appropriate attitudes and skills for providing high-quality health services [7]. Despite this awareness, very few approaches have been effective in transforming healthcare practitioners’ performance to improve quality of care in low-income countries [8].

One of the most frequently used interventions for improving healthcare services in low-income countries is training healthcare providers to improve practices [8]. However, there is significant variability in training effectiveness due to differences in trainee characteristics, programme learning outcomes and environments for knowledge transfer [8–10]. Some training interventions show significant positive changes in attitudes and practices. For example, in Pakistan, training of contraceptive service providers improved their competencies and resulted in increased uptake of services [11]. In Zambia training significantly improved the performance of providers involved in programmes designed to prevent mother to child transmission of HIV (PMTCT) [12]. Likewise, in Tanzania training of health workers resulted in significant improvements in the provision of neonatal services [13]. However, many interventions fail or perform sub-optimally [8,14]. Given that training interventions may have a transformative effect on SRHR practitioners’ attitudes and practices, there is a need for more evidence regarding their effectiveness, especially in low-income countries where poor quality of SRHR services is prevalent [1].

One of the most accepted models for assessing training interventions is Baldwin and Ford’s Transfer of Training Model [9,10,15–17]. The model defines effectiveness of training as ‘the degree to which trainees effectively apply the knowledge, attitudes and skills gained through training to the job context’ [18]. The outcome of training is influenced by individual trainee characteristics [19–21], the training design (including content, methodology and relevance) [20,22] and a conducive workplace environment that encourages trainees to use their acquired knowledge and skills [10,20,23].

This study builds on two exploratory studies that examined the association between background characteristics (individual trainee characteristics and SRHR work environment factors) and SRHR attitudes and practices among SRHR practitioners from low-income countries enrolled in an international training programme in SRHR [24,25]. The findings from the two studies suggested that SRHR knowledge gaps and SRHR attitudes (ingrained in religious or cultural beliefs) significantly influenced SRHR practitioners’ decisions during service delivery and that active SRHR knowledge seeking behaviour was significantly associated with normative SRHR practices.

The aims of the study were to 1) assess whether participating in a SRHR international training programme (ITP) changed healthcare practitioners’ SRHR knowledge, SRHR attitudes and SRHR practices and 2) examine associations between trainees’ characteristics, their SRHR work environment and *transfer of training*. We examined whether the changes differed significantly according to trainees’ baseline scores in SRHR self-rated knowledge, attitudes, knowledge seeking behaviour and practices and whether changes in SRHR knowledge and attitudes were associated with changes in SRHR practices. We hypothesised that the ITP intervention positively influenced SRHR practitioners’ knowledge, attitudes and practices.

The findings will contribute to a greater understanding of factors that impact on the effectiveness of SRHR training interventions in low-income countries.

## Methods

### Description of the international training programme

An international training programme (ITP) in SRHR which targeted healthcare practitioners from LMICs was conducted by Lund University (Sweden) between 2005 and 2018. The aim of the ITP was to develop and influence SRHR practitioners’ knowledge, attitudes and practices and facilitate more effective equitable provision of SRHR services [26]. The programme was offered at an advanced level (second cycle) equivalent to 22 ECTS (European Credit Transfer System) at Lund University [27].

The ITP covered different components of SRHR broadly [1] and specifically focused on youth sexual health, maternal and neonatal health, HIV/AIDS, cervical cancer, sexual identity and sexual violence. In addition, leadership and organisational management, health policy analysis and the use of information technology in SRHR were offered as cross-cutting themes during the training.

The training was conducted from a human rights perspective emphasising the promotion and protection of human rights, the provision of SRHR services without discrimination and fostered accountability and participation of all stakeholders [28].

The programme was grounded in socio-constructivism whereby students develop their knowledge by interaction with others from various backgrounds and cultures [29]. To enhance participatory learning and encourage sharing of experiences from diverse socio-cultural and professional backgrounds, different pedagogical approaches were used including collaborative learning, interactive lectures, case studies, value exercises, group seminars, forum discussions, peer-reviewed assignments, study visits and different forms of role-playing. Throughout the programme, trainees had access to relevant literature related to the course content [26].

The ITP was implemented in five phases. *Phase one (eight weeks)* was a preparatory phase that took place in trainees’ countries. Trainees worked as country teams to review relevant SRHR outcome indicators and their country’s demographic information. This information was later shared with other course trainees during phase two and used for planning and implementing interventions (change projects) in subsequent phases.

During *phase two (four weeks)*, trainees travelled to Lund University (Sweden) for interactive learning sessions that focused on conceptual and theoretical frameworks at the core of international SRHR policies and guidelines. Study visits to different institutions working with SRHR in Sweden were also organised. During this phase country teams were assigned supervisors, and together with their supervisors, the teams started to plan for change projects (targeted interventions aimed at addressing SRHR needs of specific populations) which were to be implemented in trainees’ home countries.

During *phase three (six months)* the trainees returned to their home countries and implemented the change projects. Lund University supervisors followed up the teams in their home countries to provide support.

*Phase four (one week*) was a results-sharing seminar. All trainees, course leaders and supervisors gathered in one of the participating countries to discuss the progress of the change projects and to plan for the projects’ sustainability.

*Phase five (six months)* was the final phase of the ITP during which change projects were fine-tuned according to the feedback received at the results seminar. Trainees were encouraged to share change project outcomes with key stakeholders in their home countries after phase five. It was assumed that participation in the ITP would contribute to positive changes in trainees’ SRHR knowledge, SRHR attitudes and SRHR practices.

### Study sample

The study sample consisted of 107 SRHR practitioners who enrolled in the ITP in 2017 and 2018. They were from Zimbabwe, Tanzania, Ethiopia, Kenya, Zambia, South Sudan, Bangladesh, Myanmar, Uganda and Liberia. The practitioners worked with different public and private sector organisations in their home countries.

### Study design

A pre- and post-intervention study design was used. The data were self-reported in questionnaires by trainees (respondents). The questionnaires were administered at the beginning of phase two of the training (*pre-training*) and approximately seven months later at the end of phase four (*post-training*). The questionnaire items yielded information about age, gender, level of education, sector of employment, area of operation in the healthcare system and the position held at training enrolment. In addition, the questionnaire was used to collect information about trainees’ self-reported SRHR knowledge, SRHR attitudes, SRHR knowledge seeking behaviour, SRHR practices, the role of religion and culture in making SRHR decisions, the importance of religion and culture in their lives and the numbers of years they had worked with SRHR. The questionnaire was pre-tested among students in an international master’s programme in public health at Lund University and some adjustments were made before administering it to the study participants.

### The conceptual framework

**Figure 1.**
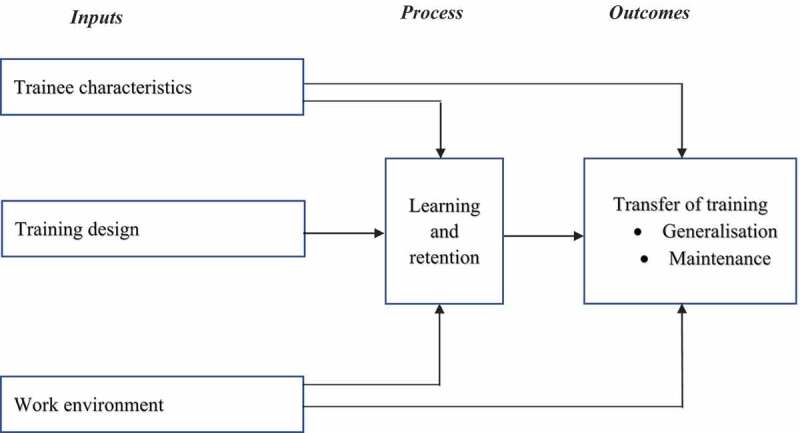
Baldwin and Ford’s transfer of training model (1988).

Baldwin and Ford’s Transfer of Training Model was used to examine the association between trainees’ individual baseline characteristics, their SRHR work environment factors and changes in their SRHR attitudes and practices after they had undergone the ITP intervention. The Model suggests that effective application of learned knowledge and acquired skills occurs when the learned knowledge and skills are generalised to one’s work environment (generalisation) and maintained over a period (maintenance). This is assumed to be facilitated by three major factors (inputs): 1) trainee characteristics which include trainee’s knowledge and self-efficacy; 2) the training programme design including the pedagogical approaches used and the relevance of training content, and 3) a work environment that encourages implementation of the acquired skills. Effective transfer is assumed to occur through two possible pathways. The training inputs (trainee characteristics, programme design and work environment) may directly influence *generalisation* and *maintenance*, or effective transfer of training can also be achieved through the *learning and retention* pathway (Figure 1) [15].

### Definition of study variables

The Knowledge, Attitudes and Practices (KAP) framework was used to develop the items in the questionnaire assessing changes in trainees’ SRHR knowledge and attitudes, and the Trans-Theoretical Model (TTM) was used to develop the items for assessing changes in trainees’ SRHR practices and knowledge seeking behaviour. The TTM describes behaviour change in terms of five distinct stages: pre-contemplation; contemplation; preparation; action and maintenance [30]. The pre-contemplation stage indicates that a person has not thought about changing their current behaviour. During the contemplation stage, one is considering changing, while a person in the preparation stage has decided to change and is taking steps to change. In the action stage, one has taken active steps in the preceding six months, and in the maintenance period a person has taken steps for more than six months. A more detailed description of the TTM and how the model was used to measure SRHR practices and SRHR knowledge seeking behaviour has been published [25]. Below we provide a brief overview of the study variables.

### Dependent variables

Effectiveness of the ITP intervention was defined as ‘improvement’ measured by change in pre- and post-training scores (delta scores) for SRHR knowledge, attitudes, knowledge seeking behaviour and practice.

### Change in SRHR attitudes

SRHR attitudes were measured by trainees’ responses to statements in the questionnaire assessing beliefs about the following aspects of SRHR: ‘abortion is a woman’s right’; ‘young people should have access to contraception’; ‘young people should have access to comprehensive sexuality education’; ‘LGBT should have equal access to HIV/STI care’; ‘men and women are affected by sexual violence equally’; ‘sexual orientation and sexual identity are human rights’, and ‘inequality is responsible for poor maternal and neonatal outcomes’. The responses to each of the statements were measured on a Likert scale of one to five whereby one indicated ‘very strong disagreement’ and five indicated ‘very strong agreement’. A composite variable for SRHR attitudes was computed by summing each trainee’s responses from each of the seven statements. Change in SRHR attitudes (delta score) was calculated for each trainee as the difference between post-training and pre-training SRHR attitudes scores. The SRHR attitudes delta scores were then dichotomised according to the mean, where scores below or equal to the mean were defined as ‘low’ (reference category) and scores above the mean defined as ‘high’.

### Change in SRHR knowledge seeking behaviour

SRHR knowledge-seeking behaviour (assessed as a practice in this study) was measured by responses indicating trainees’ intention to seek more knowledge about the following aspects of SRHR: ‘access to abortion’; ‘access to cervical cancer screening’; ‘access to contraception’; ‘access to comprehensive sexuality education’; ‘SRHR health policy’; ‘LGBT community needs’; ‘sexual coercion/sexual violence’ and ‘sexual orientation and gender identity’. The responses were rated on a Likert scale of one to five corresponding to the five stages in the TTM, where *one* indicated ‘I have not thought about seeking more information’ (precontemplation); *two* indicated ‘I’m considering seeking more information’ (contemplation); *three* indicated ‘I have decided to take steps to gain more information’ (preparation); *four* indicated ‘I have taken steps to acquire more knowledge in the last six months’ (action); and *five* indicated ‘I have taken steps to acquire more knowledge for more than six months’ (maintenance).

Each trainee’s responses to each of the eight statements were added to obtain a total score for SRHR knowledge seeking behaviour. Change in SRHR knowledge seeking behaviour (delta score) was calculated for each trainee as the difference between post- and pre-training scores. The delta scores were then dichotomised according to the mean, where scores below or equal to the mean were defined as ‘low’ (reference category) and scores above the mean as ‘high’.

### Change in SRHR practices

SRHR practices were measured by statements assessing trainee’s readiness to improve access to the following SRHR services: ‘youth access to contraception’; ‘youth access to comprehensive sexuality education’; ‘abortion services’; ‘cervical cancer screening’; ‘LGBT access to STI/HIV treatment’; support for ‘sexual violence/sexual coercion victims’, and ‘equal access to health for all’. The responses were again reported on a Likert scale of one to five corresponding to the five stages of the TTM, where *one* indicated ‘not something I have thought about’ (pre-contemplation); *two* indicated ‘is something I have thought about as important’ (contemplation); *three* indicated ‘I have decided and am taking steps to change’ (preparation); *four* indicated ‘had taken active steps in the last six months’ (action); and finally *five* indicated ‘had taken steps for more than six months’ (maintenance). Each trainee’s responses to each of the seven questions were summed to obtain a total score for SRHR practices.

Change in SRHR practices (delta score) was calculated as the difference between post- and pre-training scores for each trainee. The delta scores for SRHR practices were dichotomised according to the mean as ‘low’ if they were equal to or below the mean (reference category) and ‘high’ if they were above the mean.

### Independent variables

Trainees’ baseline individual and SRHR work environment characteristics were assessed as independent variables. Age, gender, level of education, self-rated SRHR knowledge and importance of religion or culture in one’s life were evaluated as individual characteristics.

Age was reported in two categories, ‘40 years or less’ or ‘41 years or more’ (reference category), and gender was reported as ‘male’ or ‘female’ (reference category). Education was reported as either ‘completed high school’, ‘bachelor’s degree’, ‘master’s degree’ or ‘doctorate’ and dichotomised for analysis as ‘bachelor’s degree or less’ (reference category) and ‘master’s degree or more’. Trainees also responded with either ‘yes’ (reference category) or ‘no’ to whether they considered religion or culture important in their lives. SRHR self-rated knowledge was obtained from trainees’ rating their knowledge about the different SRHR components included in ITP training: ‘comprehensive sexuality education’; ‘contraception and family planning’; ‘abortion’; ‘screening for cervical cancer’; ‘LGBT health needs’; ‘sexual orientation and gender identity’; ‘sexual violence’, and ‘SRHR policy’. They rated their knowledge on a Likert scale of one to five, where one indicated ‘very low’ knowledge and five ‘very high’. Each trainee’s total SRHR knowledge score was obtained by adding up scores obtained from each of the eight items. The difference (delta score) between post- and pre-training SRHR knowledge scores was obtained. The delta scores were then dichotomised according to the mean as either ‘low’ (reference category) for scores below or equal to the mean or ‘high’ for scores above the mean.

The influence of religion or culture on SRHR decisions, employment sector, area of operation and level of employment was assessed as the SRHR work environment. Trainees responded as either ‘yes’ (reference category) or ‘no’ to the question about whether religion or culture influenced their decisions as SRHR service providers. The area where a trainee worked was defined as area of operation and reported as: ‘local’; ‘intermediate’ or ‘national’ and dichotomised as ‘national’ (reference category) and ‘local/intermediate’. Level of employment was reported as: ‘programme officer’; ‘manager’ or ‘service provider’ and dichotomised as ‘programme officers/managers’ and ‘service providers’ (reference category). The sector of employment, in healthcare and education, was reported as either ‘public’ or ‘private’ (reference category).

Religion and culture were assumed to have a significant influence on trainees’ SRHR attitudes and practice. Hence the influence of religion and the influence of culture were analysed as the main exposure variables in this study.

### Statistical analysis

SPSS version 27 was used for data analysis. Descriptive analysis was conducted to determine the frequency and distribution of delta scores for the measured attitudes and practices across the baseline individual characteristics and SRHR work environment factors. The dependent variables were tested for assumptions of paired samples t-test and linear regression and the independent variables were tested for multicollinearity. Paired samples t-test was conducted to determine if there were statistically significant differences between trainees’ pre-training and post-training scores in self-rated knowledge, attitudes, knowledge seeking behaviour and practices. In addition, independent samples t-test was used to examine whether the effect of the ITP intervention on trainees differed significantly according to their baseline scores in SRHR self-rated knowledge, attitudes, knowledge seeking behaviour and practices. For this analysis, trainees who had obtained ‘maximum’ baseline scores were excluded (i.e. all cases with maximum score minus the mean delta score for the ‘low-baseline group’ for each scale) to address a potential ceiling effect [31]. Unadjusted association between individual characteristics, SRHR work environment factors and delta scores for SRHR attitudes and SRHR practices was determined using bivariate linear regression. Multiple linear regression was used to assess the influence of religion and the influence of culture on the delta scores for SRHR attitudes and practices after adjusting for age, gender, self-rated knowledge, education and years working with SRHR. Multiple linear regression was also used to determine whether changes in trainees’ SRHR knowledge and attitudes resulted in changes in SRHR practices after adjusting for age, gender, self-rated knowledge, education and years working with SRHR. Cases with missing data were excluded (listwise) from analysis.

### Ethics and consent

The study was approved by the Regional Ethical Review Board in Lund, Sweden (DNR 2017/823).

## Results

Of the 115 trainees enrolled in the ITP in 2017 and 2018, 107 (93%) completed the pre-and post-training questionnaire for this study.

### Trainees’ baseline characteristics

Most of the trainees were female (62.6%) and under the age of 40 years (57.9%). They had completed a bachelor’s degree or more (92%) and had been working with SRHR for seven years or less (59.8%). In addition, the majority (57%) worked with the public sector and at a national level (66.4%). Most trainees reported that religion and culture played an important role in their lives (88.8% and 69.2%, respectively). However, less than half of them indicated that religion or culture influenced their SRHR decisions (Table 1).

**Table 1.** Trainees’ baseline characteristics (*N* = 107).

		n (%)
Gender	Male	40 (37.4)
	Female	67 (62.6)
Age	Less or equal to 40 years	62 (57.9)
	40 year or more	45 (42.1)
Education	High school	8 (7.5)
	Bachelors	51 (47.7)
	Masters	40 (37.4)
	PhD	8 (7.5)
Working with SRHR	7 years or less	64 (59.8)
	8 years or more	43 (40.2)
Importance of religion	Yes	95 (88.8)
	No	12 (11.2)
Importance of culture	Yes	74 (69.2)
	No	32 (29.9)
	Missing	1 (0.9)
Influence of religion	Yes	48 (44.9)
	No	59 (55.1)
Influence of culture	Yes	44 (28.0)
	No	77 (72.0)
SRHR self-rated knowledge	Low (≤ mean)	55 (51.4)
	High (> mean)	52 (48.6)
Area of operation	Local level	20 (18.7)
	Intermediate	14 (13.1)
	National	71 (66.4)
	Missing	2 (1.9)
Employment level	Officers and managers	37 (34.6)
	Service providers	69 (64.5)
	Missing	1 (0.9)
Employment sector	Public sector	61 (57.0)
	Private sector	46 (43.0)

### Comparing trainees’ pre-and post-training scores

The assumptions of paired t-test were assessed prior to conducting the analysis. Normality of the differences between paired observations was examined using the Shapiro-Wilk test, which indicated that there was no significant departure from normality. In addition, the independence assumption was met since the paired observations were collected from the same individual independently of the others.

Paired samples t-tests indicated that there were statistically significant positive improvements in trainees’ scores for SRHR knowledge, attitudes, knowledge seeking behaviour, and SRHR practices (Table 2).

**Table 2.** Comparing trainees’ pre- and post-training scores for SRHR self-rated knowledge, SRHR attitudes, SRHR knowledge seeking behaviour and SRHR practices using paired samples t-test (*N* = 107).

Outcome variables	Measurement	Minimum-Maximum	Mean (SD)	t-value	Significance (P-value)
SRHR self-rated knowledge scores	Pre-training	10–40	27.7 (4.9)	11.9	**<0.001**
	Post- training	23–40	33.4 (3.9)		
SRHR attitudes scores	Pre- training	16–35	29.9 (3.7)	6.6	**<0.001**
	Post- training	21–35	32.4 (2.8)		
SRHR knowledge seeking behaviour scores	Pre- training	11–40	24.8 (6.6)	7.6	**<0.001**
	Post- training	10–40	30.1 (8.2)		
SRHR practices scores	Pre- training	2–35	16.7 (7.8)	6.0	**<0.001**
	Post- training	2–35	20.4 (8.4)		

The t-value (score) is the ratio between the difference between groups and the difference within the groups. A large t-value suggests that the groups are different, and a smaller t-value indicates more similarity between the two sample sets being compared.

The mean delta score for SRHR self-rated knowledge was 5.7 (SD = 4.9) with a minimum delta score of −8.0 and a maximum of 22. Likewise, the mean delta score for SRHR attitudes was 2.42 (SD = 3.8) with a minimum delta score of −7.0 and a maximum score of 19.0. The mean delta score for SRHR practices was 3.8 (SD = 6.5) with a minimum delta score of −10.0 and a maximum score of 22. For SRHR knowledge seeking behaviour, the mean delta score was 5.3 (SD = 7.2) with a minimum score of −20 and a maximum score of 24.0.

Independent samples t-tests indicated that trainees with scores below or equal to the mean in SRHR attitudes and SRHR practices benefitted the most from the ITP intervention compared to those with high scores at the beginning of the training (Table 3).

**Table 3.** Comparing trainees’ delta scores in SRHR self-rated knowledge, SRHR attitudes, SRHR knowledge seeking behaviour and SRHR practices according to their baseline scores by independent samples t-test (*N* = 107).

Baseline scores*		Mean delta scores (SD)	t-value	Significance (P-value)
SRHR self-rated knowledge scores^**1**^	≤Mean	6.1(7.5)	0.2	0.85
	>Mean	5.8 (4.3)		
SRHR attitudes^**2**^ scores	≤Mean	6.5 (5.3)	3.3	**0.001**
	>Mean	3.0 (2.4)		
SRHR knowledge seeking behaviour^**3**^	≤Mean	6.2 (7.5)	0.2	0.85
	>Mean	5.8 (4.3)		
SRHR practices^**4**^	≤Mean	5.9 (7.0)	2.7	**0.009**
	>Mean	2.4 (5.7)		

The t-value is the ratio between the difference between groups and the difference within the groups. A large t-value suggests that the groups are different, and a smaller t-value indicates more similarity between the two sample sets being compared.

*Trainees with maximum scores at baseline were excluded from analysis (i.e. all cases with maximum score minus the mean delta score for the ‘low-baseline group’ for each scale were excluded).

^**1**^excluded cases = 14.

^**2**^excluded cases = 52.

^**3**^excluded cases = 14.

^**4**^excluded cases = 8.

### Association between background characteristics and the changes in SRHR attitudes and practices

Although most trainees reported that religion and culture played important roles in their personal lives, the perceived importance of religion and culture was not significantly associated with changes in SRHR attitudes, knowledge seeking behaviours or practices (data not shown). Likewise, influence of religion and influence of culture on SRHR decisions were not significantly associated with changes in SRHR attitudes, knowledge-seeking behaviours and practices. However, high baseline SRHR knowledge scores were positively associated with improvement in attitudes scores, but not with improvements in knowledge seeking behaviour or practices (Table 4). The significant association between high baseline SRHR knowledge scores and improvement in SRHR attitudes scores remained statistically significant after controlling for the influence of religion, the influence of culture and other baseline characteristics (Table 5).

**Table 4.** Association between trainees’ baseline characteristics and changes in SRHR attitudes, SRHR knowledge seeking behaviour and SRHR practices by bivariate linear regression (*N* = 107).

	SRHR attitudes delta scores	SRHR knowledge seeking behaviour delta scores	SRHR practices delta scores
	B (95% CI)	B (95% CI)	B (95% CI)
No religious influence (Ref: Influence)	−1.05 (−2.50–0.39)	−0.15 (−2.92–2.62)	1.22 (−1.28–3.73)
No cultural influence (Ref: Influence)	0.05 (−1.42–1.53)	1.50 (−1.28–4.29)	1.39 (−1.13–3.92)
Age (Ref: 41 years or more)	−0.18 (−1.66–1.28)	0.49 (−2.30–3.28)	−.42 (−2.95–2.11)
Gender (Ref: Female)	0.27 (−1.23–1.77)	−0.81 (−3.65–2.04)	−2.03 (−4.59–.53)
SRHR self-rated knowledge (Ref: Low)	0.22 (0.36–0.07)**	−0.19 (−0.48–0.08)	.09 (−.16–.34)
Education (Ref: Bachelor’s or less)	−0.08 (−1.54–1.38)	0.26 (−2.51–3.04)	.46 (−2.05–2.98)
Working years (Ref: Less than 7 years)	−0.74 (−2.22–0.73)	0.71 (−2.09–3.52)	−1.84 (−4.37–.68)
Service providers Ref: officers/managers	0.35 (−0.39–1.09)	0.16 (−1.25–1.58)	1.64 (−.99–4.27)
Public sector employment (Ref: Private sector)	−0.35 (−1.82–1.11)	−2.45 (−5.20–0.29)	−.29 (−2.82–2.23)

Note. B=Unstandardised coefficient, P-value=**<0.01.

**Table 5.** Association between influence of religion, influence of culture and changes in SRHR attitudes, SRHR knowledge seeking behaviour and SRHR practices adjusted for other baseline characteristics by multiple linear regression (*N* = 107).

	SRHR attitudes delta scores			SRHR knowledge seeking behaviour delta scores			SRHR practices delta scores		
	Model 1 B (95% CI)	Model 2 B (95% CI)	Model 3 B (95% CI)	Model 1 B (95% CI)	Model 2 B (95% CI)	Model 3 B (95% CI)	Model 1 B (95% CI)	Model 2 B (95% CI)	Model 3 B (95% CI)
No religious influence (Ref: Influence)	−1.26 (−2.85 0.31)	−1.30 (−2.91–0.30)	−0.97 (−2.57–0.63)	−0.86 (−3.87–2.14)	−0.81 (−3.86–2.24)	−0.62 (−3.76–2.51)	0.86 (−1.92–3.53)	1.05 (−1.67–3.79)	1.17 (−1.63–3.98)
No cultural influence (Ref: Influence)	0.56 (−1.04–2.16)	0.56 (−1.05–2.17)	0.60 −0.97–2.18)	1.84 (−1.19–4.88)	1.84 (−1.22–4.91)	1.99 (−1.09–5.08)	1.07 (−1.68–3.83)	1.11 (−1.63–3.86)	0.79 (−2.00–3.59)
Age (Ref: 41 years or more)	-	−0.19 (−1.69–1.31)	−0.19 (−1.72–1.33)	-	0.63 (−2.24–3.49)	0.26 (−2.73–3.27)	-	−0.16 (−2.73–2.41)	1.88 (−2.51–2.89)
Gender (Ref: Female)	-	0.43 (−1.11–1.98)	0.14 (−1.39–1.66)	-	−0.94 (−3.87–1.99)	−1.22 (−4.22–1.77)	-	−2.20 (−4.83–0.43)	−2.22 (−4.91–0.47)
SRHR self-rated knowledge (Ref: Low)		-	0.21** (0.35–0.05)		-	−0.22 (−0.52–0.07)		-	0.04 (−0.23–0.30
Education (Ref: Bachelor’s or less)		-	0.16 (−1.31–1.64)		-	0.38 (−2.51–3.27)		-	−1.25 (−3.98–1.47)
Working years (Ref: Less than 7 years)		-	−0.61 (−0.21–0.89)		-	0.78 (−2.15–3.73)		-	−1.75 (−4.43–0.92)

B=Unstandardised coefficient, P-value=**<0.01.

Model 1. Adjusted for influence of religion and influence of culture.

Model 2. Adjusted for model 1 plus age and gender.

Model 3. Adjusted for model 1 and model 2 plus, SRHR self-rated knowledge, education level and working years with SRHR.

### Testing the knowledge-attitude-practice hypothesis

When changes in SRHR knowledge and attitudes scores were examined as potential predictors of changes in SRHR practice scores and adjusted for knowledge seeking behaviour and other covariates, the changes in SRHR knowledge and attitudes did not show any statistically significant association with changes in SRHR practices. However, high improvement in SRHR knowledge seeking behaviour was significantly associated with high improvement in SRHR practices (Table 6).

**Table 6.** Association between changes in SRHR self-rated knowledge, changes in SRHR attitudes and changes in SRHR practices adjusted for change in SRHR knowledge seeking behaviour and other covariates by multiple linear regression (*N* = 107).

	SRHR practices delta scores		
	Model 1 B (95% CI)	Model 2 B (95% CI)	Model 3 B (95% CI)
SRHR self-rated knowledge (Ref: low delta scores)	−0.01 (−0.26–0.25)	−0.01 (−0.26–0.24)	0.13 (−0.24–0.27)
SRHR attitudes (Ref: low delta scores)	0.22 (−0.12–0.55)	0.20 −0.13–0.53)	0.22 (−0.12–0.56)
SRHR knowledge seeking behaviour (Ref: low delta scores)	**0.28** (0.11–0.46)**	**0.28** (0.11–0.46)**	**0.28** (0.10–0.0.46)**
Age (Ref: 41 years or more)	-	0.23 (−2.29–2.73)	−0.34 (−2.94–2.25)
Gender (Ref: Female)	-	−1.82 (−4.29–0.65)	−1.77 (−4.31–0.78)
8 years or more working years (Ref: Less than 7 years)	-	−1.88 −4.35–0.59)	−1.99 (−4.47–0.49)
No religious influence (Ref: Influence)	-	-	1.55 (−1.08–4.19)
No cultural influence (Ref: Influence)	-	-	0.53 (−2.10–3.16)
Service providers (Ref: Officers and managers)	-	-	1.76 (−0.89–4.40)

B=Unstandardised coefficient, P-value=* <*0.05*, **<0.01, ***< 0.001.

Model 1. Adjusted for SRHR self-rated knowledge, SRHR attitudes, knowledge seeking behaviour delta scores.

Model 2. Adjusted for model 1 plus age and gender.

Model 3. Adjusted for model 1 and model 2 plus influence of religion, influence of culture, level of employment.

## Discussion

The trainees who completed the SRHR training programme showed statistically significant positive improvements in their SRHR knowledge, attitudes and practices. Trainees’ baseline characteristics (e.g. age, gender, level of education) were not significantly associated with changes in the measured attitudes and practices. Trainees with low baseline scores in SRHR attitudes and practices seem to have benefitted more from the ITP intervention. Nonetheless, improving knowledge and attitudes did not seem to have a direct effect on improving SRHR practices.

Similar to what was found at the baseline survey [25], most individual characteristics and SRHR work environment factors were not associated with SRHR attitudes and practices after the training intervention. Although more years working with SRHR and no influence of religion were significantly associated with normative SRHR attitudes and SRHR practices at baseline [25], no such findings were obtained after the intervention. However, high baseline SRHR knowledge scores were associated with improvements in SRHR attitudes scores after the ITP intervention. Few studies have examined the association between SRHR knowledge and SRHR attitudes among health practitioners in low- and middle-income countries. Nevertheless, low knowledge of contraception (a component of SRHR) among community health workers in Kashmir [32] and in Nigeria [33] was associated with negative attitudes towards contraceptive use, and high knowledge of gender-based violence among health workers in Brazil was associated with more favourable attitudes towards victims of gender-based violence [34]. This may imply that interventions that improve health practitioners’ SRHR knowledge may result in improved SRHR attitudes and contribute to a higher quality of services in low-income countries.

The study utilised Baldwin’s and Ford’s Transfer of Training Model to assess the effect of an international training programme on SRHR practitioners’ knowledge, attitudes and practices [15]. The model facilitates the evaluation of the association between trainee characteristics, workplace factors and training effectiveness. The findings of this study suggest that even though interventions like the ITP may improve trainees’ knowledge and attitudes, their impact on the desired practices (generalisation and maintenance) may be influenced by other factors beyond the acquisition of new knowledge or changing attitudes. It has been suggested that the relationship between changing attitudes and changing behaviours may be offset by different processes underlying long-standing practices [35,36]. In fact, Triandis [37] argues that changing attitudes are unlikely to change long-standing acquired behaviours. It is proposed that changing long-standing behaviour requires context-specific interventions that target behaviour mechanisms [35].

In this study, improving SRHR knowledge seeking behaviour scores was significantly associated with improvement in SRHR practices. The course design was grounded in socio-constructivism where participants have ample opportunities to exchange knowledge, thoughts and feelings with each other and teachers [29] and experiential learning [38] with the learning activities involving active engagement, concrete experiences, reflection and abstract conceptualisation. Active knowledge seeking is an intentional behaviour typically exhibited by self-regulated learners [39]. Learners’ eagerness to plan and control their own learning has been linked to effective attainment of educational goals [40,41] and may have a life-long effect on decision-making and behaviour [42,43]. Current developments in global health require health practitioners to learn more by themselves and continuously. Training interventions may facilitate development of lifelong learning strategies and transform health practitioners’ behaviours if they harness the potential of developing self-regulated learning strategies. Learner-centred, experiential and inquiry-based educational approaches that have shown great potential in developing skills for lifelong learning could be prioritised among behaviour change interventions involving healthcare practitioners [40].

In this study, we did not investigate the effect of the ITP intervention on the individuals who started the training with maximum scores, ‘the ceiling effect’, based on the assumption that they had no room for improvement [31]. However, it is plausible that such individuals benefitted from the ITP intervention in ways that have not been explored in this study.

### Methodological considerations

Most participants in our study had a bachelor’s degree (or more) and were working at a national level in their respective healthcare systems. This may have introduced a selection bias in the study sample. Therefore, the findings may not be generalisable to all healthcare practitioners in low-income countries. However, most of our study participants were employed in the public sector, which is the biggest provider of health services in many low-income countries, and hence the findings may apply to similar settings. In addition, the study lacked a comparison group, and therefore, the observed changes in SRHR attitudes and practices should be interpreted cautiously. Further research with an experimental study design is needed to examine the observations drawn from this study.

This study had two major limitations. One was the small sample size which could have resulted in significant underestimation of the association between trainee background characteristics and the effectiveness of the ITP intervention. The other weakness was the use of self-reported data. Even though self-reported data provides us with participants’ perceptions and beliefs, it is prone to bias including social desirability bias and recall bias, both of which may have resulted in over- or under-estimation of the measured associations. To minimise social desirability bias, participants were assured of confidentiality and anonymity at the time of data collection and the questionnaire was self-administered. A short follow-up period for this study could have minimised recall bias. However, on the other hand, the induction time between intervention and effect might be longer than the follow-up time, and thus lead to an underestimation of the effect.

The main strengths of the study are the prospective assessment of the trainees to measure changes in SRHR attitudes and practices over time and the use of Baldwin and Ford’s Transfer of Training Model that provided an appropriate conceptual framework to assess the effectiveness of the ITP intervention. The model has been used extensively in different research settings and found to be reliable and valid [10,15].

### Implications for training of health workforce

Results from this study support the use of training as an effective intervention for improving healthcare practitioners’ SRHR attitudes and practices in LMICs. Furthermore, they highlight the role of knowledge seeking behaviour as a strong predictor for improving health practitioners’ SRHR practices. Future training interventions could focus on improving trainees’ knowledge-seeking behaviour to facilitate transfer of training and improve quality of SRHR services.

## Conclusion

The findings indicate that the ITP was effective in improving trainees’ self-rated scores for SRHR knowledge, attitudes and behaviours (practices). The strongest association was found between improvement in SRHR knowledge seeking behaviour and the improvement in SRHR practices. This suggests that behaviour intention may have a central role in promoting fair open-minded SRHR practices among healthcare practitioners in low-income countries.

## Supplementary Material

Supplementary table (S1)

## Paper context

In low-income countries, healthcare practitioners’ biases and preconceptions can negatively impact access to sexual and reproductive health and rights (SRHR) services. This study showed that an international training programme for SRHR practitioners improved their knowledge-seeking behaviour and practices. Behavioural intentions may play a central role in promoting fair, open-minded, non-biased delivery of SRHR services in low-income countries.
