# Supplementary material for: Assessing the effectiveness of a sexual and reproductive health and rights training programme in changing healthcare practitioners’ attitudes and practices in low-income countries
Source: Glob Health Action. 2023 Jul 17;16(1):2230814. doi: 10.1080/16549716.2023.2230814 (PMC10353321; doi:10.1080/16549716.2023.2230814)
Supplement: Supplementary table (S1) [file ZGHA_A_2230814_SM2804.pdf]

Supplementary table (S1). Distribution of delta scores (changes in) SRHR attitudes, SRHR knowledge seeking behaviour and SRHR practices according to baseline characteristics by chi-square test (N=107).

		SRHR attitudes delta scores			SRHR knowledge seeking behaviour delta scores			SRHR practices delta scores		
		≤ mean n (%)	> mean n (%)	p-value	≤ mean n (%)	> mean n (%)	p-value	≤ mean n (%)	> mean n (%)	p-value
Gender	Male	23 (57.5)	17 (42.5)		19 (47.5)	21 (52.5)		19 (47.5)	21 (52.5)	
	Female	37 (55.2)	30 (44.8)	0.81	34 (50.7)	33 (49.3)	0.75	38 (56.7)	29 (43.3)	0.35
Age	Less or equal to 40 years	35 (56.5)	27 (43.5)		29 (46.8)	33 (53.3)		35 (56.5)	27 (43.5)	
	40 year or more	25 (55.6)	20 (44.4)	0.92	24 (53.3)	21 (46.7)	0.50	22 (48.9)	23 (51.1)	0.43
Education	Bachelor's degree or less	31 (52.5)	28 (47.5)		30 (50.8)	29 (49.2)		35 (59.3)	24 (40.7)	0.16
	Master's degree or more	29 (60.4)	19 (39.6)	0.41	23 (47.9)	25 (52.1)	0.76	22 (45.8)	26 (54.2)	
Working with SRHR	7 years or less	33 (51.6)	31 (48.4)		32 (50.0)	32 (50.0)		33 (51.6)	31 (48.4)	
	8 years or more	27 (62.8)	16 (37.2)	0.25	21 (48.8)	22 (51.2)	0.91	24 (55.8)	19 (44.2)	0.66
Influence of religion	Yes	**21 (43.8)**	**27 (56.3)**		24 (50.0)	24 (50.0)	0.93	26 (54.2)	22 (45.8)	
	No	**39 (66.1)**	**20 (33.9)**	**0.02**	29 (49.2)	30 (50.8)		31 (52.5)	28 (47.5)	0.86
Influence of culture	Yes	25 (56.8)	19 (43.2)		23 (52.3)	21 (47.7)		25 (56.8)	19 (43.2)	
	No	35 (55.6)	28 (44.4)	0.89	30 (47.6)	33 (52.4)	0.63	32 (50.8)	31 (49.2)	0.54
SRHR self-rated knowledge	Low (≤ mean)	28 (50.9)	27 (49.1)		24 (43.6)	31 (56.4)		27 (49.1)	28 (50.9)	0.37
	High (> mean)	32 (61.5)	20 (38.5)	0.27	29 (55.8)	23 (44.2)	0.21	30 (57.7)	22 (42.3)	
Area of operation	Local/intermediate	17 (50.0)	17 (50.0)		17 (50.0)	17 (50.0)		17 (50.0)	17 (50.0)	
	National	43 (60.6)	28 (39.4)	0.30	35 (49.3)	36 (50.7)	0.95	40 (56.3)	31 (43.7)	0.54
Employment level	Officers/managers	19 (51.4)	18 (48.6)		18 (48.6)	19 (51.4)		**27 (73.0)**	**10 (27.0)**	
	Service providers	40 (58.0)	29 (42.0)	0.51	34 (49.3)	35 (50.7)	0.95	**30 (43.5)**	**39 (56.5)**	**0.004**
Employment sector	Public sector	32 (52.5)	29 (47.5)		27 (44.3)	34 (55.7)		31 (50.8)	30 (49.2)	0.55
	Private sector	28 (60.9)	18 (39.1)	0.38	26 (56.5)	20 (43.5)	0.21	26 (56.5)	20 (43.5)	
